# The Role of the Immune Microenvironment in Bone Regeneration

**DOI:** 10.7150/ijms.61080

**Published:** 2021-09-21

**Authors:** Ning Yang, Yao Liu

**Affiliations:** 1 Department of Pediatric Dentistry, School and Hospital of Stomatology, China Medical University, Shenyang, China.; 2 Liaoning Provincial Key Laboratory of Oral Diseases, Shenyang, China.

**Keywords:** immune microenvironment, bone regeneration, immune cell, immune cytokine, osteoimmunology

## Abstract

Bone is an active tissue, being constantly renewed in healthy individuals with participation of the immune system to a large extent. Any imbalance between the processes of bone formation and bone resorption is linked to various inflammatory bone diseases. The immune system plays an important role in tissue formation and bone resorption. Recently, many studies have demonstrated complex interactions between the immune and skeletal systems. Both of immune cells and cytokines contribute to the regulation of bone homeostasis, and bone cells, including osteoblasts, osteoclasts, osteocytes, also influence the cellular functions of immune cells. These crosstalk mechanisms between the bone and immune system finally emerged, forming a new field of research called osteoimmunology. Therefore, the immune microenvironment is crucial in determining the speed and outcome of bone healing, repair, and regeneration. In this review, we summarise the role of the immune microenvironment in bone regeneration from the aspects of immune cells and immune cytokines. The elucidation of immune mechanisms involved in the process of bone regeneration would provide new therapeutic targets for improving the curative effects of bone injury treatment.

## Introduction

Bone is composed of osteoblasts, osteoclasts, osteocytes, and other basic elements. As a dynamic organ, there is a balance between bone formation and bone resorption and there are complex interactions between the immune and skeletal systems. Bone and immune cells not only co-exist in the bone marrow (BM) cavity, but also have common progenitor cells and share a variety of regulatory molecules. Immune cells participate in the regulation of bone homeostasis; conversely, bone cells also influence the proliferation and differentiation of immune cells. Therefore, the term 'osteoimmunology' was coined to highlight the two-way communication between the bone and immune systems 1. Additionally, the immune system plays an important role in tissue repair and regeneration. The immune microenvironment is crucial in the healing, repair, and regeneration of bone tissue, which determines the ability of bone tissue to regenerate 2. In this review, we summarise the role of the immune microenvironment in bone regeneration from the aspect of immune cells and cytokines, which is helpful in elucidating the immune mechanisms involved in the bone regeneration process and provides a new therapeutic target for improving the curative effect of bone injury treatment.

## Bone metabolism

The skeleton is an extremely dynamic organ that undergoes remodeling throughout life to maintain bone strength and mineral homeostasis by constantly renewing the bone matrix 3. Physiological bone remodelling is a dynamic equilibrium occurring as a result of a series of well-orchestrated biological events, all regulated by complex interactions between the various cell types that are resident in bone. The main cells involved in bone remodelling are osteoblasts and osteoclasts 4. The functions of these cells are closely coupled, sequentially carrying out the absorption of old damaged bone and the formation of new bone 4. In addition, osteocytes, acted as mechanical sensors, also play an important role in the process of bone remodelling, while bone lining cells initiate bone remodelling through matrix degradation 5, 6.

The bone remodelling cycle consists of five sequential phases: activation, resorption, reversal, formation, and termination. In the activation phase, unknown triggering signals are induced on the cell surfaces of osteoblasts and/or bone lining cells, providing the site of bone remodelling onset, where osteoclasts are recruited and fused to form mature osteoclasts. In the following resorption phase, osteoclasts dissolve the inorganic matrix by creating an acidic microenvironment and degrade the organic matrix with specific enzymes. Next is the reversal phase, in which, with the completion of bone resorption, osteoclasts undergo apoptosis, macrophage-like reverse cells migrate to the absorbed lacuna and clear the debris left by the osteoclasts; subsequently, osteoblasts are summoned to the resorption lacuna to synthesise new bone matrix and then mineralise it to fill the resorption lacuna in the formation phase. Once mineralisation is complete, osteoblasts undergo apoptosis, change into bone lining cells, or are buried within the bone matrix and eventually differentiate into osteocytes in the termination phase. The resting bone surface environment is maintained until the next wave of remodelling is initiated 7, 8.

Therefore, bone coupling involves the interaction of a wide range of cell types and control mechanisms, including signals from immune cells and signaling taking place within the osteoblast lineage. It has been shown, that macrophages maintain osteoblast differentiation, which may be related to the Oncostatin M secreted by activated macrophage 9, 10. T cells can mediate the anabolic action of parathyroid hormone, which stimulates bone formation and triggers osteoblast's secretion of pro-hematopoietic factors like IL-6 and IL-6R 11, 12. And the recent identification of Wnt1 as a B-cell product in bone marrow that promotes bone formation 13.

## Role of immune cells in bone regeneration

Tissue damage triggers an immediate immune response, which usually lasts for days or even weeks. The initial immune response is mainly composed of the innate immune system, including neutrophils, mast cells, monocytes, and macrophages. The later immune response is mainly composed of an adaptive immune system, including T and B cells. The impact of immune cells on the bone regeneration have two main aspects: The anabolic function of acute inflammation during bone regeneration and the catabolic function of chronic inflammation during bone resorption. For example, acute inflammation leading to the production of chemokines that accelerates bone formation in early bone fracture healing by stimulating the proliferation and osteoblastic differentiation of mesenchymal progenitor cells 14, 15. In contrast, chronic inflammation suppresses the production of factors that stimulates osteoblastic cells to form new bone, thereby inhibiting osseous coupling and promoting osteolytic lesion formation during periodontitis 15, 16.

### Neutrophils

As the most abundant leukocytes in mammalian blood, neutrophils are well recognised as one of the major players of the innate immune system. Upon tissue injury, neutrophils are usually the first to be recruited and migrated to the injured site. Neutrophils clear invasive pathogenic microorganisms, initiate an acute inflammatory response, and enhance host defence 17. A large number of clinical cases and animal experiments have confirmed that neutrophil aggregation and infiltration is seen in bone injury and plays a key role in the early stages of bone repair. In a mouse model of bone fractures, the authors noted that depletion of neutrophils led to impaired bone healing after fracture 18. Furthermore, they also observed increased levels of inflammatory cytokines as well as altered monocyte and macrophage recruitment, indicating a crucial role for neutrophils in initiating the bone repair process 18. However, some studies have reported that bacteria produce endotoxin-lipopolysaccharide during trauma to stimulate neutrophils to express receptor activator of nuclear factor kappa B ligand (RANKL), which activates osteoclasts to exert bone resorption 19, 20. Moreover, in contrast to CD3^+^ T lymphocytes, activated neutrophils only express the membrane protein RANKL and do not secrete soluble RANKL, suggesting that neutrophils activate osteoclast function only through inter-cellular contact 20, 21. In osteoporosis induced by long-term glucocorticoid treatment and chronic gout, neutrophils directly inhibit bone formation by affecting the function of osteoblasts 22, 23. Additionally, neutrophils have also been confirmed to play an important role in bone lesions in animal experiments of osteoarthritis and aggressive periodontitis 24, 25. In summary, neutrophils not only perform a function as immune cell, but also regulate bone homeostasis in the initial stage of bone injury.

### Macrophages

Macrophages, derived from monocytes, are ubiquitous in tissues, act as phagocytes to prevent pathogens from invading, and effectively remove necrotic tissue and cell debris. Upon bone injury, macrophages secrete release various cytokines, chemokines, and growth factors to initiate the recruitment of fibroblasts, MSCs, and osteoprogenitor cells from their local niches 26. Additional macrophage-derived inflammatory mediators and growth factors guide the proliferation, differentiation and extracellular matrix production of recruited MSCs and osteoprogenitor cells with additional growth factors being released from the remodeling extracellular matrix, which plays an important role in tissue repair and regeneration 27. Under the stimulation of endogenous or exogenous factors, macrophages are transformed into two phenotypes: M1 and M2. M1 macrophages are generally pro-inflammatory, whereas M2 macrophages are anti-inflammatory 28. In some cases, the M1 phenotype can be reversed to M2, *in vitro* and *in vivo*, and vice versa 29. A growing number of reports have demonstrated macrophages' central role in osteogenesis. Macrophages support osteoblast differentiation and proliferation through the release of cytokines including BMP-2, BMP-4, and TGF-β1 30, 31. In contrast to other tissue regeneration, macrophages participate in every phase of bone injury, healing, and regeneration, but the specific mechanism is not clear. Using a mouse model of femoral fracture, it has been found that macrophages are not involved in the early stage of fracture healing, but prevent the ultimate endochondral ossification by inhibiting the formation of hard callus 32. Further studies have shown that M2 macrophages are crucial in the ossification phase of bone regeneration. By increasing the ratio of M2 macrophages through interleukin 4 (IL-4) and IL-13, bone regeneration can be significantly enhanced 32. Vi *et al.* found that compared with wild-type mice, macrophage-deficient mice display decreased numbers of trabeculae, and reduced bone regeneration ability after fracture, suggesting that M2 macrophages promote osteoblast differentiation 33. M1 macrophages play an important pro-inflammatory role in the early stage of bone injury, which helps to resist the invasion of bacteria, viruses, and other pathogens, promotes antigen presentation, activates the immune response, and initiates tissue regeneration.

In addition, studies have also shown that all macrophage subtypes have the capacity to promote MSC mediated osteogenesis but their relevance may change during different physiological conditions. Loi *et al.* examined the impact of sequential modulation of macrophage phenotype on the osteogenic ability of pre-osteoblasts. All of the macrophage phenotypes M0, M1 and M2 increased the osteogenic ability of MC3T3 cells. Furthermore, modulation of macrophage polarization from M1 to M2 *via* IL-4 treatment after 72 h of M1-MC3T3 co-culture further enhanced bone formation, suggesting that transient inflammatory phase followed by M2 dominated regenerative phase might lead to optimal bone formation 34. However, sustained, chronic, M1-macrophage mediated inflammation reduce the curative effect of bone fracture healing by inhibiting neovascularisation and prolonging the initial inflammatory response 35. And the continued production of pro-inflammatory cytokines of M1 results in bone resorption *via* increasing osteoclast activity and suppression of bone formation by osteoblasts 36. Thus, the transformation ratio of M1 to M2 macrophages in different stages of bone regeneration is particularly important, with brief acute inflammation being beneficial and chronic inflammation highly detrimental to bone healing.

### Dendritic Cells

Currently, dendritic cells (DCs) are the most functional antigen-presenting cells known. Major histocompatibility complex II (MHC II) are highly expressed on the surface of DC membranes, which effectively activates the immune function of T cells and initiates the immune response 37. In the physiological state, DCs do not exist in bone tissue or adjacent BM matrix, and there is no obvious abnormality of the skeletal system in DC-deficient mice 38. However, in a study of rheumatoid arthritis (RA), it was found that a large number of immature and mature DCs gathered in the active lesion site of the synovium and distributed around the adjacent bone tissue 39. Chemotactically aggregated DCs interact with T cells through the RANK-RANKL signalling pathway to activate Th1 inflammatory-type responses and improve osteoclast activity 40. DCs are present in oral gingival tissue. In chronic periodontitis, DCs aggregate into the periodontal lesion tissue to destroy periodontal bone tissue by forming aggregates with T cells 41. Therefore, it is believed that DCs may indirectly affect inflammation-related bone loss by activating and regulating T cell function.

Both DCs and osteoclasts are derived from monocyte/macrophage precursor cells. Granulocyte macrophage colony-stimulating factor (GM-CSF) promotes monocyte/macrophage precursor cells to differentiate into DCs and inhibit osteoclast formation. The specific molecular mechanism is related to the c-Fos signal 42. Co-stimulation of macrophage colony-stimulating factor (M-CSF) and RANKL induces human peripheral blood-derived DCs to differentiate into osteoclasts under *in vitro* inductive conditions 43, suggesting that M-CSF and GM-CSF have opposite effects on the regulation of DC and osteoclast differentiation. Alnaeeli *et al.* have found that mouse CD11c^+^ DCs co-cultured with CD4^+^ T cells differentiate into functional osteoclasts and that systematic infusion of CD11c^+^ DCs delays the repair and regeneration of mouse parietal bone defects 44. These results clearly indicate that DCs have the potential to differentiate into osteoclasts, directly regulate the bone resorption process, and then affect bone remodelling and regeneration.

### Innate lymphoid cells

Innate lymphoid cells (ILCs) are the most recently identified subset of innate lymphocytes. The members of the ILC family, mainly composed of natural killer cells, ILC1, ILC2, ILC3, and lymphoid tissue inducer cells. ILCs can be classified in three principal groups according to their transcription factor expression and cytokine secretion 45. Group 1 ILCs (ILC1s) consist of NK cells and ILC1, which produce IFN-γ. ILC2s secrete IL-13 and IL-5, while ILC3s consists of lymphoid tissue inducer cells and ILC3 which secrete IL-22 and IL-17 46. In the last few years, different reports highlighted the important contribution of ILCs to bone homeostasis. The research shows that ILCs are present in periodontal tissues of mice 47. Further, periodontal tissues of the ligature-induced murine model of periodontitis display significant increases in each subset of ILCs with the effect more marked for ILC2s 47. Importantly, periodontal tissues of human subjects obtained from sites affected by periodontitis show remarkable similarities to those of the murine model 47, 48. Furthermore, RANKL expression was detected on a fraction of the ILC1s suggesting a possible role of ILCs to directly contribute to inflammatory osteolysis 48. However, Omata *et al.* found that ILC2 suppressed osteoclast formation through the release of cytokines, such as IL-4 and IL-13, and adoptive transfer of ILC2 completely abrogated ovariectomy-induced bone loss 49. To sum up, the role of ILCs in bone homeostasis is controversial and needs further study.

### T Cells

T cells are important lymphocytes in the adaptive immune system, are derived from hematopoietic stem cells, develop and mature in the thymus, and then migrate to the peripheral lymphoid organs. Many studies have confirmed that T cells produce a series of cytokines and growth factors, and play a key role in the process of bone remodelling and regeneration. According to different T cell receptors, T cells are divided into two categories: αβ T cells and γδ T cells. αβ T cells are further subcategorised into CD4^+^ and CD8^+^ T cells, which have dual functions of promoting and inhibiting regeneration. γδ T cells are a small subset of T cells, which are considered to promote regeneration, but the mechanism is not clear 50, 51. Therefore, the regulatory role of T cells in bone regeneration may be related to T cell subsets, cytokines, and local factors.

#### T help cell 1 (Th1)/Th2 Cells

Under antigen stimulation, naive T cells differentiate into Th1 and Th2 cells. Th1 cells mainly secrete interferon γ (IFN-γ), IL-2, and tumour necrosis factor α (TNF-α) to eradicate intracellular pathogens. Th2 cells are involved in the activation of B cells and elimination of extracellular pathogens by the secretion of IL-4, IL-5, IL-6, and IL-10 52. At present, the role of Th1 and Th2 cells in bone regeneration is still controversial. Sato *et al.* found that IFN-γ and IL-4 produced by Th1 and Th2 cells inhibit osteoclast differentiation and promote bone formation by degrading TNF receptor associated factor 6 (TRAF-6) 53. Previous studies have demonstrated that Th1 cells are the main source of RANKL. Th1 cells mainly promote osteoclast precursor cell differentiation and activate osteoclast function through TNF-α, which indirectly leads to bone tissue absorption and destruction 54. In addition, under conditions of oestrogen deficiency or inflammation, the osteoclastogenic effect of Th1 cells is dominant 55.

#### Th17 Cell

Th17 cells are a type of CD4^+^ T cells that have been discovered in recent years. Under the stimulation of transforming growth factor β (TGF-β) and IL-6, naive T cells differentiate into Th17 cells, which mainly secrete IL-17, IL-22, and IL-26, and promote bone resorption 56. IL-17 produced by Th17 cells induces the expression of RANKL on the surface of osteoclast precursors, bone marrow stromal cells, and osteoblasts, thus promoting osteoclast formation and inhibiting osteoblast differentiation and function 57. Th17 cells directly promote the bone resorption process by secreting increasing amounts of active RANKL compared to Th1 cells 53. IL-17 also indirectly enhances bone resorption by recruiting and activating other immune cells to up-regulate levels of TNF-α and IL-1 in bone tissue 58. In summary, Th17 cells, known as osteoclastogenesis-supporting T cells, and their pro-inflammatory cytokine IL-17, play a negative regulatory role in bone regeneration.

#### T regulatory Cells (Treg)

T regulatory cells (Treg) are a subset of T cells with immunosuppressive functions, which secrete cytokines, such as IL-4 and TGF-β, maintain autoantigen immune tolerance, and homeostasis of the immune system. Treg play an important positive regulatory role in bone wound healing and regeneration. Zaiss *et al.* found that transgenic mice with elevated level of Treg display higher bone mineral density and lower bone resorption in their long bones than wild-type mice 59. In a study of the repair of calvarial defects in mice, systematic injection of Treg was found to effectively reduce the levels of inflammatory factors in the local area of trauma, improve the osteogenic ability of transplanted stem cells, and finally achieve functional bone regeneration 54. *In vitro* studies have also found that Treg cells directly up-regulate the function of osteoblasts and improve the osteogenic differentiation ability of osteoblast precursor cells, bone marrow mesenchymal stem cells (BMMSCs) 60. This mechanism may be related to the CD39-CD93-adenosine receptor (AdoR) signalling pathway 60. Other studies have discovered that Treg interact with CD8^+^ T cells to up-regulate the production of WNT10b, which acts on stromal cells and osteoblasts to promote bone formation 61. Moreover, Treg are able to downregulate the differentiation and function of osteoclasts: Treg directly inhibit the differentiation of peripheral blood-derived monocytes into osteoclasts 62; and they also inhibit osteoclast function by secreting cytotoxic T-lymphocyte associated protein 4 (CTLA4) to activate the CD80/CD86 receptor of osteoclast precursors 63. Uncontrolled inflammation after tissue injury can lead to impaired healing and tissue remodeling. In the process of bone repair, Tregs are recruited to the damaged site to facilitate inflammation resolution and to regulate immunopathologic reactions after injury 64. For instance, Tregs indirectly promote tissue regeneration by controlling neutrophil behavior and macrophage polarization 65, 66. Moreover, as immunosuppressive cells, Treg effectively inhibit over-activated T cells in inflammatory lesions, such as tissue trauma, inflammation, and tumours, induce immune tolerance, and reduce levels of Th1, Th17, and pro-inflammatory cytokines in local tissue, thus promoting bone regeneration. For instance, it has been demonstrated that Tregs facilitate MSC-based bone regeneration by inhibiting CD4^+^ T-cells, which secrete IFN-γ and TNF-α 54.

### B Cells

B cells are derived from hematopoietic stem cells in the BM and enter the spleen and lymph nodes after maturation. B cells proliferate and differentiate into plasma cells under the stimulation of antigens. As an important source of antibody synthesis and secretion, B cells are an important part of the adaptive immune system. In addition to immune function, the differentiation and maturation of B cells are closely related to bone cells. B cells differentiate and mature in the BM cavity, where BMMSCs provide a stable microenvironment for B cell differentiation. An abnormal BM phenotype leads to B cell differentiation and dysfunction 67. Although the role of B cells during normal bone remodelling appears minimal, activated B cells play an important role in inflammation-related bone diseases. In multiple myeloma, B cells promote bone resorption by directly up-regulating the expression of RANKL and blocking the activity of runt-related transcription factor 2 (RUNX2)/core-binding factor alpha 1 68, 69. Plasma cells derived from malignant B cells secrete proteases such as sclerostin and Dickkopf WNT signalling pathway inhibitor 1 to inhibit osteoblast differentiation 70. B cells in RA mice express high levels of the osteoblast inhibitors C-C motif chemokine ligand 3 and TNF, and inhibit mesenchymal precursor cell differentiation into osteoblasts by activating extracellular signal-regulated kinase and NF-κB signalling pathways, while B cell depletion attenuates bone erosion and osteoblast inhibition in RA mice 71. In addition, in osteoporosis caused by oestrogen deficiency, B cells secrete RANKL to promote osteoclast function, which is one of the important mechanisms of decreased bone mineral density 72.

B cells and plasma cells are the main sources of osteoprotegerin (OPG) in bone tissue. B cells antagonistically block the effect of RANKL by secreting OPG and promoting bone tissue regeneration. In an inflammatory environment, activated T cells promote B cell to product OPG *via* the CD40/CD40L signalling pathway, which has a promoting effect on bone tissue regeneration 73. In an *in vitro* osteoclast culture system, TGF-β secreted by B cells induces osteoclast apoptosis; TGF-β further promotes B cells to produce OPG, suggesting that B cells inhibit osteoclast differentiation 74. In summary, the role of B cells in bone regeneration is still controversial, and its specific role may depend on local pathological conditions.

## Role of immune cytokines in bone regeneration

Immune cells play a key role in the process of tissue injury, healing, and regeneration, and cytokines are important effectors of immune cells. Cytokines may play different roles in promoting or inhibiting bone tissue regeneration, which may be related to the type, concentration, expression time of cytokines, as well as local environment.

### TNF-α

TNF-α is a transmembrane protein mainly produced by monocytes, binding to its receptor (TNFR1, TNFR2): TNF-α binds to TNFR1 to initiate NF-κB signalling pathways, as well as binds to TNFR2 to activate T cells *via* mitogen-activated protein kinase (MAPK) 75. At present, TNF-α is believed to inhibit osteogenesis, promote bone resorption, and negatively regulate the bone homeostasis of many chronic and inflammatory diseases 76. Recent studies indicate that osteoblast lineage cells, including PDL cells, that have osteoblastic characteristics are the primary source of RANKL 77. RANKL produced by periodontal ligament and bone lining cells is required for orthodontic tooth movement and formation of osteoclast 78. Also RANKL produced by osteocytes is critical for inflammation induced bone loss in a periodontitis model 79. The increased expression of TNF-α leads to nuclear factor-kappa B activation in periodontal ligament cells, osteoblasts, and osteocytes, which has negative impact on bone formation and expression of bone-producing factors such as fibroblast growth factor-2, TGF-β1, bone morphogenetic protein-2, and bone morphogenetic protein 16, 80. However, recent studies have indicated that vesicular RANK, which is secreted from the maturing osteoclasts, binds osteoblastic RANKL and promotes bone formation by triggering RANKL reverse signaling, indicating that osteoblastic RANKL functions also as a coupling signal acceptor that recognizes vesicular RANK 81. Additional, TNF-α suppresses osteoblast differentiation by inhibiting classical WNT signalling, and inhibits osteogenic process by up-regulating the expression of the SMAD ubiquitin regulatory factor in osteoblasts 82, 83. In addition, TNF-α induces osteoclast precursor cells to chemotaxis to bone resorption sites and differentiate into mature osteoclasts 84.

Interestingly, some researchers believe that TNF-α promotes bone healing and regeneration. The level of TNF-α reached its peak 24 h after fracture, which was helpful in recruiting BMMSCs involved in bone regeneration into the injured tissue. The second peak appears 4 weeks after fracture, which is essential for endochondral ossification, suggesting that TNF-α may be a cytokine that marks the beginning of tissue healing 85. Thus, the role of TNF-α in bone healing and regeneration is regulated by many factors, and its effect mainly depends on the concentration and the differentiation state of the osteocytes, as well as on the exposure time 86.

### IL-1

IL-1 is produced by activated monocytes-macrophages and exists in the form of IL-1α and IL-1β. IL-1 is a key factor in osteoclast differentiation and formation, and its mechanism is related to the RANKL/RANK signalling pathway. IL-1α may promote osteoclast differentiation by stimulating the secretion of prostaglandin E2 (PGE2) 87. Systematic injection of IL-1α into mice causes hypercalcemia and increases the number of osteoclast precursors and osteoclasts in the bone marrow 88. IL-1β enhances bone resorption by promoting osteoclast adherence to the surface of calcified bone trabeculae 89. In addition, IL-1β induces peripheral blood mononuclear cells to pass through the vascular endothelium, gather and differentiate into osteoclasts 90. A large amount of IL-1β is released during bone injury and binds to IL-1R1/MyD88 receptors, which promotes osteoclast function while reducing MSC proliferation, migration, and differentiation, thus inhibiting bone regeneration 91.

In the process of fracture healing, IL-1 not only has a synergistic effect with TNF-α, but also serves as the basis for the function of TNF-α 92. In the mouse fracture healing model, the concentration of IL-1 increases sharply after fracture and peaks after 24 h, during which time IL-1 promotes the release of pro-inflammatory factors, such as IL-6 and PGE2, and promotes angiogenesis and cartilage callus formation to stabilise the fracture site 93. The second peak appears 3 weeks after fracture, when IL-1 promotes protease activity to degrade injured tissue and promotes bone remodelling and regeneration 93.

### IL-6

IL-6 is a pleiotropic immune cytokine expressed and secreted by osteoblast cell lines and osteoclasts, which is essential for normal bone development and bone regeneration. Compared with wild-type mice, IL-6 knockout mice show symptoms, such as decreased bone mineral density, a reduced number of bone trabeculae, decreased fracture healing ability, and significantly delayed mineralisation and callus maturation 94. The specific mechanism is related to IL-6, which regulates the differentiation of osteoblasts and osteoclasts, and stimulates the release of vascular endothelial growth factor to promote vascularisation. In addition, *in vitro* experiments show that IL-6 treatment promotes the expression of the osteogenic proteins RUNX2 and osteocalcin, and promotes osteoblast differentiation 95. All the above studies suggest that IL-6 plays an important role in promoting bone regeneration.

In contrast to other cytokines, such as IL-1 and TNF-α, IL-6 does not play a regulatory role in the whole process of bone tissue injury, healing, and regeneration. The expression level of IL-6 peaked on the first day after fracture and decreased significantly after operation or seven days after fracture. Volpin *et al.* also confirmed that IL-6 levels in patients with different types of fracture returns to normal, six months after injury, which suggests that IL-6 is crucial in promoting tissue healing and osteogenic regeneration in the early stage of bone tissue injury and can thus be used as an early marker of fracture healing 96, 97.

### IL-17

IL-17 is a pro-inflammatory cytokine that has been discovered in recent years, and is mainly secreted by Th17 cells. After binding to its receptor, IL-17 promotes inflammatory development and activates the immune response, which plays an important role in inflammation and autoimmune diseases 98. Although IL-17 as the main effector of “osteoclastogenesis supporting Th17 cells”, however its exact effect on osteoclasts is not clear. Some studies have reported that IL-17 up-regulates the activity of osteoclasts in animal models of arthritis, but the specific mechanism may be related to the indirect effects of other cytokines, such as IL-1, TNF-α, and RANKL 99. Recent studies have found that IL-17 is crucial in the regulation of MSCs. Huang *et al.* found that IL-17 stimulates the formation of BMMSC clones in humans and mice, suggesting that IL-17 derived from T cells may act as a growth factor for MSCs 100. Other studies have also shown that IL-17 effectively stimulates the proliferation, migration, and osteogenic differentiation of MSCs, but inhibits adipogenic differentiation 101, 102. Interestingly, IL-17 inhibits the differentiation and maturation of mouse myoblast line cells into muscle cells and promotes differentiation into osteoblasts 103. Moreover, IL-17 acts synergistically with bone morphogenetic protein 2 (BMP-2) to enhance new bone formation 104. In summary, the role of IL-17 in bone regeneration is controversial and needs further study.

### IFN-γ

IFN-γ was first found in RA, and then entered the field of osteoimmunology; it inhibits the excessive formation of osteoclasts and has an osteoprotective effect 105. At present, there is no consensus regarding the effect of IFN-γ on osteoclasts. IFN-γ inhibits RANKL signalling by activating the ubiquitin proteasome to accelerate TRAF-6 degradation, directly affects osteoclast precursors, and inhibits osteoclast formation and maturation 106, suggesting that IFN-γ is the central link of T cells inhibiting osteoclast formation induced by RANKL. Although there is evidence that IFN-γ inhibits osteoclasts *in vitro*, its effect *in vivo* is more complex. In the treatment of osteosclerosis with IFN-γ, IFN-γ induces the expression of MHC II molecules *in vivo*, promotes antigen presentation, and directly inhibits the formation of osteoclasts by targeting mature osteoclasts, leading to the process of osteoclast formation and bone absorption 107.

In addition, IFN-γ has a regulatory effect on osteoblasts and their progenitor cells. MSCs cultured in osteo-inductive conditions *in vitro* secrete a large amount of IFN-γ to promote osteogenic differentiation 108. Compared with wild-type mice, IFN-γ receptor knockout mice exhibit lower structural parameters of cortical and trabecular bone, showing an osteoporotic phenotype 109. On the other hand, the synergistic effect of IFN-γ and TNF-α inhibits the osteogenic and adipogenic differentiation of MSCs through the NF-κB signalling pathway 110. IFN-γ activates indoleamine, which regulates the metabolism of MSCs through the tryptophan/kynurenine pathway, thus inhibiting the differentiation of MSCs into osteoblasts 111. In summary, IFN-γ plays an important role in the regulation of bone homeostasis and bone regeneration.

### Additional Cytokines

In addition to the inflammatory cytokines described above, there are other cytokines that exert an effect on MSCs during the process of bone regeneration and remodeling. Recent studies have identified that the IL-12 cytokine family, consisting of pro-inflammatory IL-12 and IL-23 and the anti-inflammatory IL-27 and IL-35 cytokines, play an important role in bone regeneration 112. The proinflammatory cytokines of IL-12 family, namely, IL-12 and IL-23, indirectly inhibited BMMSC differentiation by stimulating CD4^+^ T cells to increase IFN-γ and IL-17 113. Anti-inflammatory cytokine IL-27, was found to have inhibitory effects on osteoclastogenesis and osteoblast apoptosis, thus promotes bone formation 114. Moreover, IL-35, the most recently discovered IL-12 family member, is a direct inhibitor of osteoclastogenesis 115.

## Future prospects of applied osteoimmunology

Achieving satisfactory reconstruction of bone remains an important goal in orthopedic and dental conditions such as bone trauma, osteoporosis, arthritis and periodontitis. An acute inflammatory response is crucial at the onset of bone repair, while an adaptive immune response has important implications during late bone remodeling. During the initial phase of bone healing, macrophages and neutrophils respond by releasing a variety of cytokines, chemokines, and growth factors. As part of the adaptive immune response, T and B cells and their inflammatory cytokines play an important role in regulating bone regeneration 116. A well-controlled inflammatory response can be beneficial to bone repair, but can be destructive to bone tissue when the inflammation is unregulated 117. For example, reduced levels of bone matrix molecules and the osteoblast transcription factors in peri-implantitis 118. In another model of rheumatoid arthritis, resolution of inflammation was accompanied by bone regeneration 119. Taken together, the control of inflammation is indispensable for an anabolic microenvironment that supports bone formation and repair.

The challenge remains as to how these cellular mechanisms can be modulated to the benefit of the patient. From a clinical point of view, by modifying the surface qualities of biomaterials while maintaining stability and longevity, it may cause favorable responses of immune cells 120, thus giving immunomodulatory properties to transplanted materials, which is expected to provide new strategies for future orthopedic surgery, trauma surgery and implant repair.

In addition, the monoclonal antibody therapy has marked a milestone in the treatment of bone damage caused by immune-related diseases such as rheumatoid arthritis. For example, Tocilizumab (target: IL-6R) and Adalimumab (target: TNF-α) are approved very successful therapeutic drugs for rheumatoid arthritis 121, 122. However, in view of the complex relationship between bone and immune system, it seems meaningful to find the best therapeutic target so as to open up new strategies for therapies based on molecular and cellular principles of osteoimmunology.

## Conclusions and prospects

The repair and regeneration of bone tissue is a complex process involving the participation of a variety of cells and is regulated by many factors. Immune cells and immune cytokines play critical roles in regulating the balance of bone formation and bone resorption. However, the immunomodulatory mechanism of bone regeneration is still unclear. Currently, increasing evidence indicates that regulating the immune microenvironment is a promising therapeutic target to promote bone tissue functional regeneration. At the same time, we still face many problems, such as how to select correct immune cells and immune factors as targets for bone regeneration therapy to establish effective immune microenvironment, and how to combine immune microenvironment regulation with bone tissue engineering. It is believed that further studies on the roles of immunology in bone regeneration will provide new and effective targets for the treatment of bone tissue defects.

## Figures and Tables

**Figure 1:**
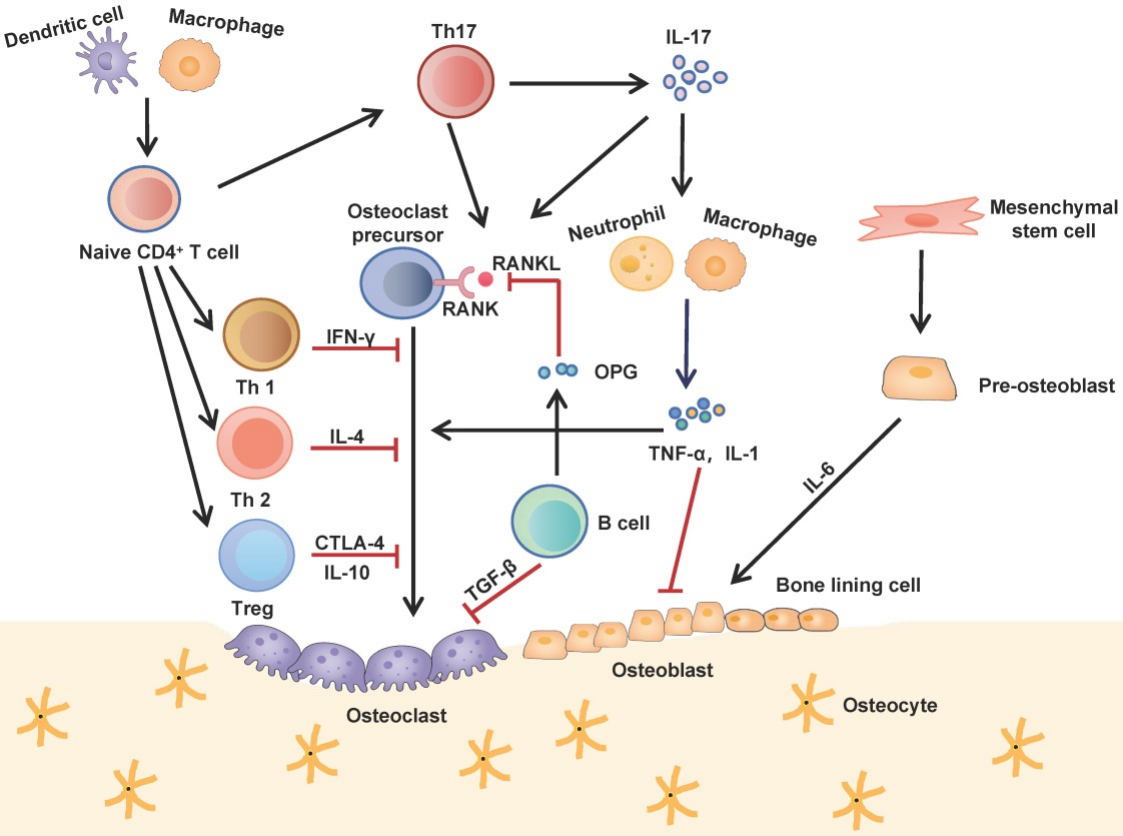
** Interactions between the immune and skeletal systems.** The T cell subsets (Th1, Th2, Th17 and Treg) play an important role in bone repair and regeneration. Th17 cells, known as osteoclastogenesis-supporting T cells, which secret IL-17 to upregulate RANKL expression and induce inflammatory cytokines such as TNF-α and IL-1 from innate immune cells. These cytokines further activate osteoclast precursor cells and inhibite osteoblast function. Contrarily, Th1, Th2 and Treg inhibit osteoclastogenesis by secreting cytokines INF-γ, IL-4, CTLA-4 and IL-10 respectively. B cells antagonistically block the effect of RANKL by secreting OPG and induce osteoclast apoptosis by secreting TGF-β.

## Abbreviations

BM: bone marrow
RANKL: receptor activator of nuclear factor kappa B ligand
IL-4: interleukin 4
DCs: dendritic cells
MHC II: major histocompatibility complex II
RA: rheumatoid arthritis
GM-CSF: granulocyte macrophage colony-stimulating factor
M-CSF: macrophage colony-stimulating factor
ILCs: Innate lymphoid cells
ILC1s: Group 1 ILCs
Th1: T help cell 1
IFN-γ: interferon γ
TNF-α: tumour necrosis factor α
TRAF-6: TNF receptor associated factor 6
TGF-β: transforming growth factor β
Treg: T regulatory Cells
BMMSCs: bone marrow mesenchymal stem cells
AdoR: adenosine receptor
CTLA4: cytotoxic T-lymphocyte associated protein 4
RUNX2: runt-related transcription factor 2
OPG: osteoprotegerin
MAPK: mitogen-activated protein kinase
MSCs: mesenchymal stem cells
PGE2: prostaglandin E2
BMP-2: bone morphogenetic protein 2
